# Inhibition of Renin-Angiotensin System Reverses Endothelial Dysfunction and Oxidative Stress in Estrogen Deficient Rats

**DOI:** 10.1371/journal.pone.0017437

**Published:** 2011-03-29

**Authors:** Lai Ming Yung, Wing Tak Wong, Xiao Yu Tian, Fung Ping Leung, Lai Hang Yung, Zhen Yu Chen, Xiaoqiang Yao, Chi Wai Lau, Yu Huang

**Affiliations:** 1 Institute of Vascular Medicine, Li Ka Shing Institute of Health Sciences, School of Biomedical Sciences, The Chinese University of Hong Kong, Hong Kong, China; 2 Department of Biochemistry, The Chinese University of Hong Kong, Hong Kong, China; University of Valencia, Spain

## Abstract

**Background:**

Estrogen deficiency increases the cardiovascular risks in postmenopausal women. Inhibition of the renin-angiotensin system (RAS) and associated oxidative stress confers a cardiovascular protection, but the role of RAS in estrogen deficiency-related vascular dysfunction is unclear. The present study investigates whether the up-regulation of RAS and associated oxidative stress contributes to the development of endothelial dysfunction during estrogen deficiency in ovariectomized (OVX) rats.

**Methodology/Principal Findings:**

Adult female rats were ovariectomized with and without chronic treatment with valsartan and enalapril. Isometric force measurement was performed in isolated aortae. The expression of RAS components was determined by immunohistochemistry and Western blotting method while ROS accumulation in the vascular wall was evaluated by dihydroethidium fluorescence. Ovariectomy increased the expression of angiotensin-converting enzyme (ACE), angiotensin II type 1 receptor (AT_1_R), NAD(P)H oxidase, and nitrotyrosine in the rat aorta. An over-production of angiotensin II and ROS was accompanied by decreased phosphorylation of eNOS at Ser^1177^ in OVX rat aortae. These pathophysiological changes were closely coupled with increased oxidative stress and decreased nitric oxide bioavailability, culminating in markedly impaired endothelium-dependent relaxations. Furthermore, endothelial dysfunction and increased oxidative stress in aortae of OVX rats were inhibited or reversed by chronic RAS inhibition with enalapril or valsartan.

**Conclusions/Significance:**

The novel findings highlight a significant therapeutic benefit of RAS blockade in the treatment of endothelial dysfunction-related vascular complications in postmenopausal states.

## Introduction

Menopause is a risk factor for cardiovascular diseases as estrogen deficiency is known to impair cardiovascular function and metabolism [1]. Loss of estrogen-dependent cardiovascular protection diminishes endothelial function, and may involve activation of the renin-angiotensin system (RAS). Clinical and animal studies indicate an inverse association between estrogen and the RAS activation [2], [3], [4], [5], [6]. Endothelial dysfunction caused by reduced bioavailability of nitric oxide (NO) and/or elevated formation of reactive oxygen species (ROS) in the vascular wall, sets into motion in a sequence of events leading to the development of cardiovascular complications [7], [8]. Endothelial dysfunction occurs during estrogen deficiency [9], [10] and estrogen improves endothelial function in postmenopausal women [11]. Ample evidences suggest a critical involvement of the RAS in the initiation of endothelial dysfunction [12], [13]. Angiotensin II elicits several harmful effects on vascular wall through angiotensin type 1 receptor (AT_1_R) including vasoconstriction, vascular smooth muscle cell (VSMC) proliferation, ROS generation, and endothelial cell apoptosis [14], [15], [16]. Cardiovascular protection can thus be achieved by either inhibiting the synthesis of angiotensin II or by blocking the binding of angiotensin II to AT_1_R.

Hypertension and osteoporosis are the two important age-related disorders in postmenopausal women. Angiotensin II infusion accelerates osteoporosis in ovariectomized (OVX) rats [17]. Although there is some concern that use of AT_1_R blockers could exacerbate postmenopausal osteoporosis, a recent animal study did not confirm this as chronic treatment of OVX rats with valsartan did not accelerate OVX-induced bone loss [18]. Instead, treatment of hypertensive mice with an ACE inhibitor reduces osteroporosis and hypertension [19]. Thus, blockade of angiotensin II may be an effective therapeutic approach to prevent osteoporosis and treat hypertension in postmenopausal women usually afflicted by both age-related conditions. Limited clinical data also shows that AT_1_R blockers improve endothelial dysfunction after menopause and treatment with candesartan lowers blood pressure [20] and ameliorates endothelial dysfunction [21] in postmenopausal women.

Despite reported therapeutic benefit of RAS inhibition in hypertension, the precise role of the activation of the RAS-oxidative stress axis in the induction and maintenance of endothelial dysfunction in the estrogen-deficient state remains unclear. Therefore, the present study investigates the hypothesis that the activation of the RAS and associated oxidative stress mediate endothelial dysfunction during estrogen deficiency and chronic treatment with enalapril (ACE inhibitor) or valsartan (AT_1_R blocker) could restore the impaired endothelial function in estrogen-deficient OVX rats, an animal model widely employed to mimic menopause.

## Results

### Basic parameters

Rats had an increased body weight 12 weeks after OVX and this gain was unaltered by enalapril or valsartan. The reduced uterine weight and serum oestradiol level in OVX rats were unaffected by chronic RAS blockade (Table 1). OVX caused a small but insignificant increase in blood pressure compared with sham-operated rats (p>0.05). Chronic treatment with enalapril or valsartan slightly lowered blood pressure, but did not affect OVX-induced increase in heart weight or decrease in the uterine weight and estrogen level (Table 1). The ratio of heart weight over body weight was comparable in the four treatment groups (Table 1). OVX elevated plasma levels of total cholesterol, triglyceride, HDL and non-HDL, while valsartan or enalapril treatment produced varied effects on these parameters (Table 1).

**Table 1:** Basic Parameters.

	Control	OVX	OVX + Ena	OVX + Val
**Basic Parameters**				
Body weight (BW) (g)	237.5±4.8	351.7±6.0^a,***^	337.0±4.9	345.0±8.5
Systolic blood pressure (mmHg)	101.0±2.9	105.9±2.0	97.9±1.4^b,**^	99.6±1.8^b,*^
Heart weight (HW) (g)	0.95±0.01	1.35±0.01^a,**^	1.28±0.01	1.29±0.04
Uterus weight (g)	0.49±0.06	0.09±0.01^a,***^	0.07±0.01	0.07±0.01
HW/BW (%)	0.40±0.005	0.38±0.025	0.38±0.008	0.36±0.012
**Plasma biomarkers**				
Plasma estrogen (pg/ml)	21.7±1.11	5.63±0.35^a,***^	6.48±0.28	5.67±0.53
Total cholesterol (mg/dl)	85.8±1.9	122.0±1.6^a,***^	123.2±2.0	138.6±3.1^b,***^
Triglyceride (mg/dl)	81.8±0.4	112.4±2.2^a,***^	100.3±5.9	106.0±2.0
HDL (mg/dl)	51.2±0.8	60.1±0.5^a,***^	77.4±2.2^b,***^	72.1±1.0^b,***^
Non-HDL (mg/dl)	34.7±2.2	61.9±1.7^a,***^	45.8±2.7^b,***^	66.2±3.8
Non-HDL/HDL ratio	0.68±0.05	1.03±0.03^a,***^	0.92±0.06	0.60±0.05^b,***^

Basic parameters measured in control, OVX rat with and without chronic treatment with enalapril (OVX+Ena) or valsartan (OVX+Val) include body weight, blood pressure, heart weight and uterine weight. Plasma parameters include oestrogen and various lipids. Results are means±SEM of six animals. Statistical significance between (a) control versus OVX and (b) OVX versus treatment is indicated by * p<0.05, ** p<0.01, and *** p<0.001.

### Ovariectomy inhibits endothelium (NO)-dependent but not -independent relaxations

**Figure 1:**
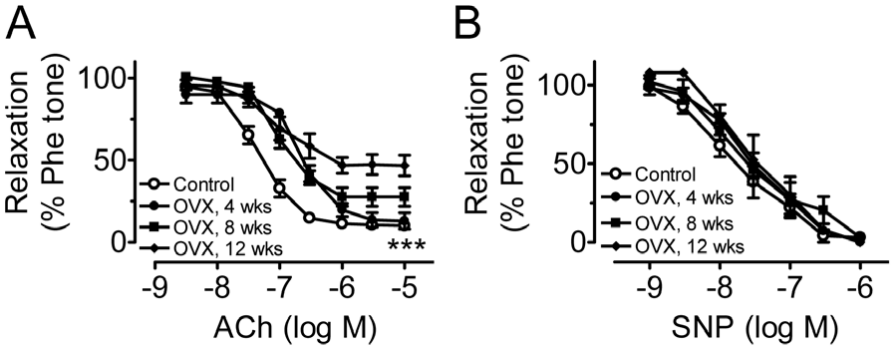
Ovariectomy impairs endothelium-dependent relaxation. Time-dependent reduction of endothelium-dependent relaxations induced by acetylcholine (ACh, A) but not by sodium nitroprusside (SNP, B) in phenylephrine (Phe)-contracted ring with endothelium following ovariectomy. Results are means±SEM of 6–8 experiments. Statistical significance between control and OVX curves is indicated by *** p<0.001.

Acetylcholine-induced endothelium-dependent relaxations were impaired progressively following OVX with time-dependent reductions in the maximum response (Figure 1A, Table 2). By contrast, SNP-induced endothelium-independent relaxations were similar at different time points after OVX (Figure 1B), indicating that the sensitivity of aortic smooth muscle cells in response to NO was unaltered in estrogen-deficient rats.

**Table 2:** pD_2_ and E_max_ (%) for acetylcholine-induced relaxations.

	Initial tone (g)	pD_2_	E_max_ (%)	n
Control (12 wks)	0.60±0.03	7.39±.07	92.7±1.9	6
OVX (4 wks)	0.92±0.07^a,**^	6.60±0.10^a,***^	93.1±2.9	6
OVX (8 wks)	0.93±0.11^a,**^	7.02±0.10^a,*^	76.7±3.0^a,**^	6
OVX (12 wks)	1.00±0.06^a,***^	7.04±0.18^a,*^	55.0±3.5^a,***^	8
**Acute treatment**				
OVX (12 wks)	1.00±0.06	7.04±0.18	55.0±1.9	8
+Losartan (Los)	1.10±0.04	7.20±0.10	84.3±2.7^b,***^	6
+Apocynin (Apo)	0.93±0.20	7.17±0.13	86.7±3.3^b,***^	6
+Los+Apo	0.95±0.16	7.11±0.08	89.5±2.3^b,***^	6
+Tiron+DETCA	0.90±0.18	7.26±0.07	90.1±1.9^b,***^	6
**Chronic treatment**				
Control	0.65±0.12	7.37±0.07	89.0±1.9	6
OVX	1.12±0.09^a,**^	6.88±0.11^a,*^	50.1±2.2^a,***^	8
OVX+Enalapril	0.81±0.08^b,*^	7.23±0.12	79.4±3.2^b,***^	8
OVX+Valsartan	1.07±0.06	7.15±0.10^b,*^	83.6±2.8^b,***^	8

Initial tension developed by phenylephrine, pD_2_ and E_max_ (%) for acetylcholine-induced relaxations in aortae from different groups. Results are means±SEM of 6–8 experiments. Statistical significance between (^a^) control versus OVX and (^b^) OVX versus acute or chronic treatment is indicated by *p<0.05, **p<0.01, and ***p<0.001.

### AT_1_R inhibitor and ROS scavengers acutely ameliorate OVX-related endothelial dysfunction

**Figure 2:**
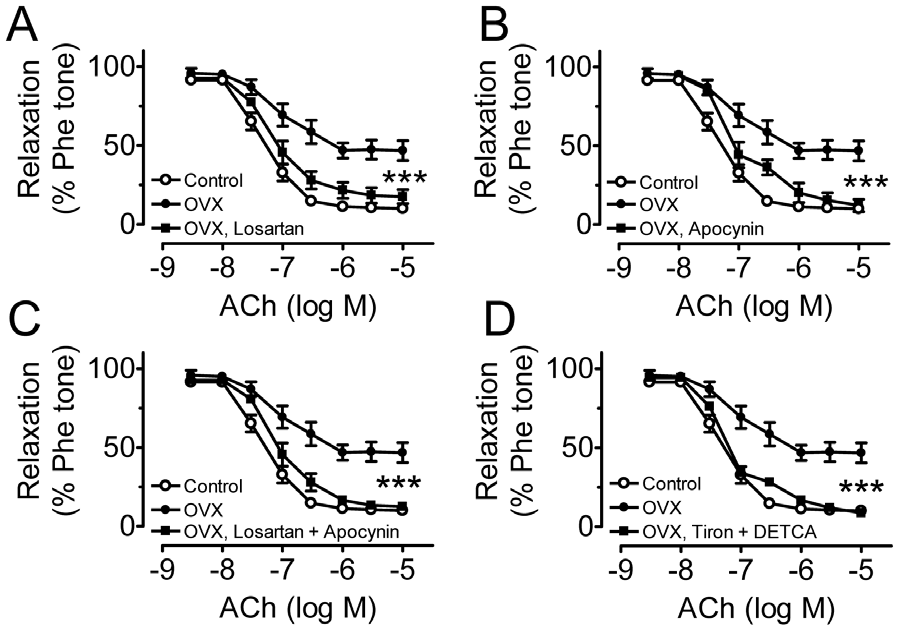
AT_1_R inhibitor and ROS scavengers acutely ameliorate OVX-related endothelial dysfunction. Inhibitory effects of 30-min treatment with 3 µmol/l losartan (A), 100 µmol/l apocynin (B), 3 µmol/l losartan plus 100 µmol/l apocynin (C), and 1 mmol/l tiron plus 100 µmol/l DETCA (D) on ACh-induced relaxations. Results are means±SEM of 6–8 experiments. Statistical significance between OVX and drug-treated OVX is indicated by *** p<0.001.

The impaired endothelial NO-mediated relaxations in OVX rats were reversed acutely by 30-min treatment with losartan (Figure 2A). Acute exposure to apocynin also restored the impaired relaxations (Figure 2B). A combined treatment with losartan and apocynin did not result in additional improvement of the relaxations (Figure 2C). The important role of ROS in the impaired endothelial function was further supported by a complete restoration of relaxations by ROS scavengers, tiron plus DETCA (Figure 2D). The ACh-induced relaxations under pharmacological treatments were abolished by 100 µmol/l N^G^-nitro-L-arginine methyl ester (L-NAME) (data not shown).

### Chronic RAS blockade prevents endothelial dysfunction

**Figure 3:**
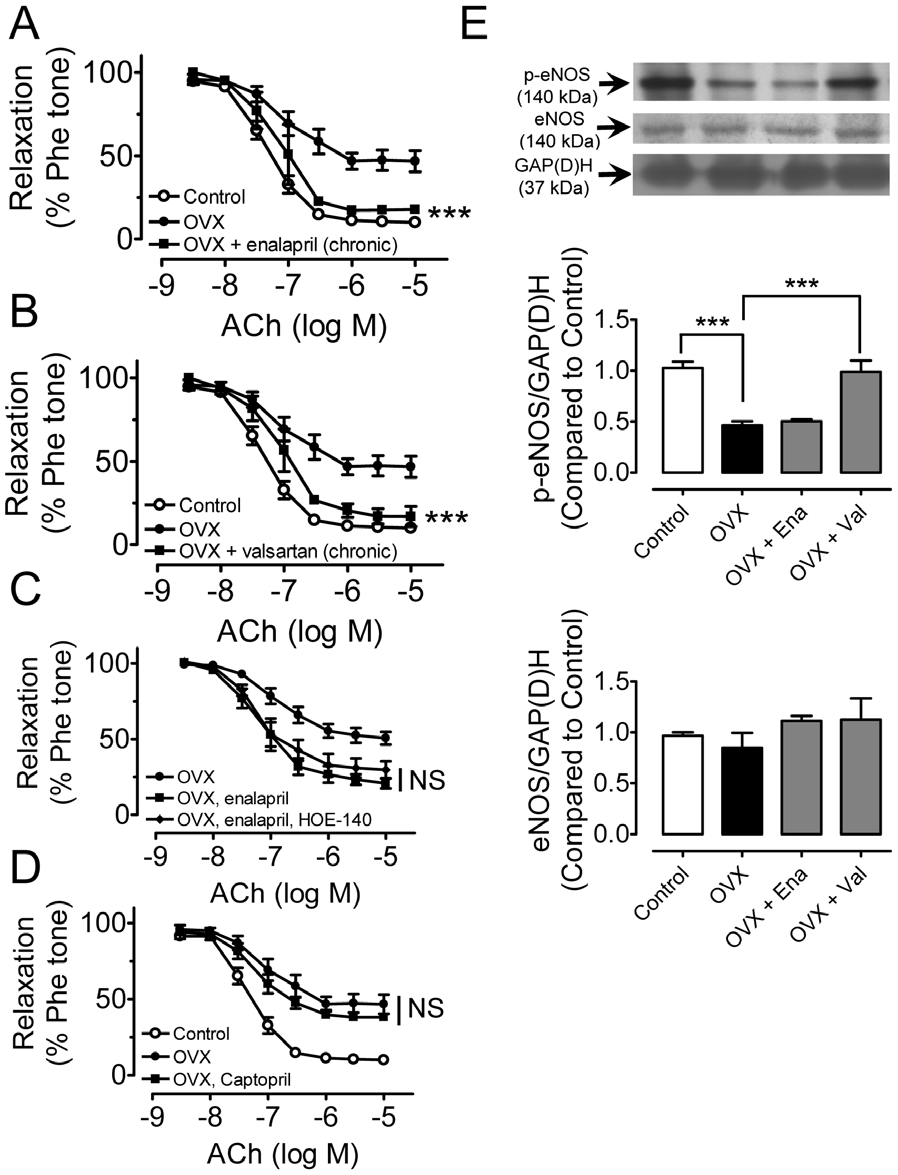
Chronic RAS blockade prevents endothelial dysfunction. Concentration-response curves for ACh in aortae from control, OVX rats and OVX rats treated with enalapril (A, OVX+Ena) or valsartan (B, OVX+Val). (C) Lack of effect of HOE-140 on relaxations in aortae from OVX+Ena group. (D) Lack of effect of captopril on relaxations in OVX rats. (E) Phosphorylated levels of eNOS (p-eNOS) at Ser^1177^ in response to 1 µmol/l ACh and the total eNOS in aortae. Results are means±SEM of 6–8 experiments. Intensities were normalized to GAP(D)H and expressed relative to control. Statistical significance between OVX and treatment group is indicated by *** p<0.001. NS, no significance.

Chronic treatment of OVX rats with enalapril (Figure 3A) or valsartan (Figure 3B) reversed the impaired acetylcholine-induced relaxations. Chronic treatment with valsartan (but not enalapril) restored the phosphorylation of eNOS at ser^1177^ in the presence of acetylcholine, which was reduced in OVX rat aortae, while total eNOS expressions were not altered among all the groups (Figure 3E). The improved relaxations in enalapril-treated OVX rats was unaffected by 30-min treatment with 100 nmol/l HOE-140, a bradykinin type-2 receptor antagonist (Figure 3C). In addition, acute treatment with the ACE inhibitor captopril did not improve the impaired relaxation in OVX rat aortae (Figure 3D), suggesting that acute ACE inhibition in isolated aortas from OVX rats can not reduce the downstream ROS production.

### ACE expression and angiotensin II levels in the vascular wall

**Figure 4:**
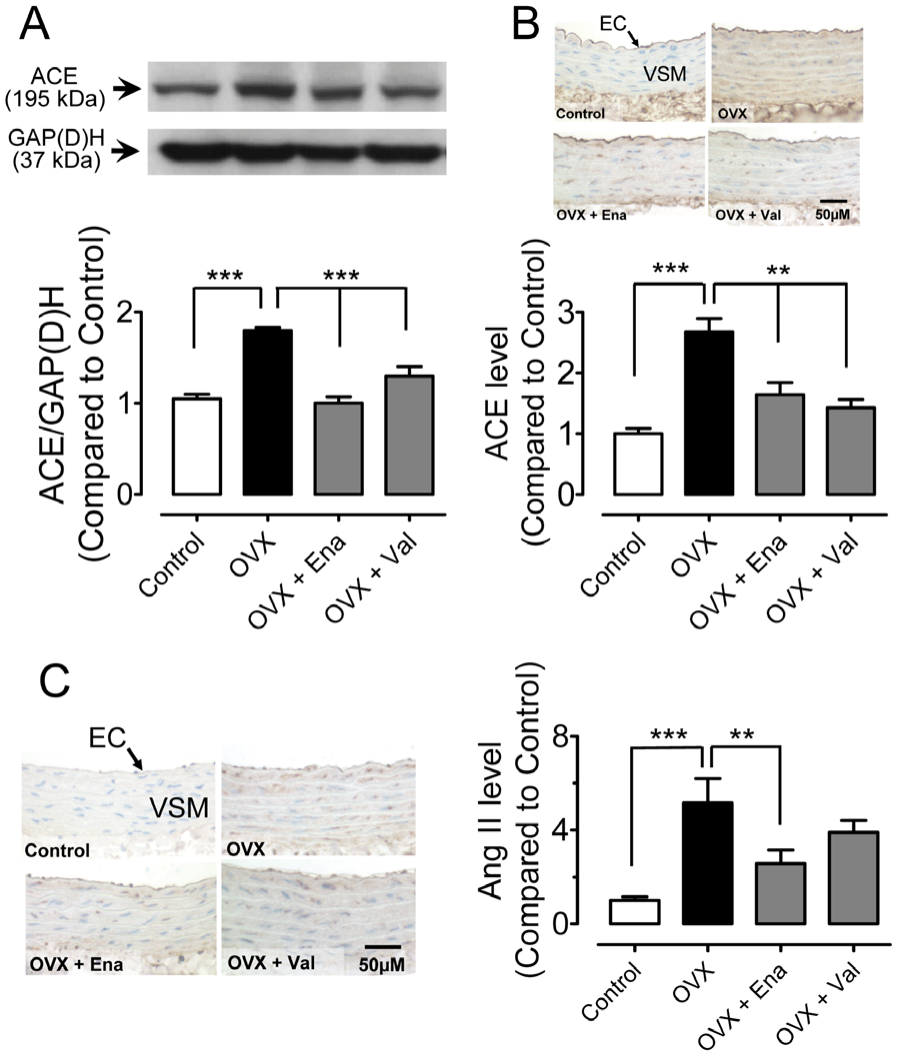
Chronic RAS blockade reduces ACE expression and angiotensin II levels after ovariectomy. Effects of chronic treatment of OVX rats with enalapril or valsartan on the protein level of angiotensin-converting enzyme (ACE) revealed by Western blot (A) and immunohistochemistry (B). Immunohistochemical staining of angiotensin II in the aortic vascular wall (C). Sections are counterstained with hematoxylin. Magnification ×400. Results are means±SEM of 4–6 experiments. Statistical significance is indicated by ** p<0.01 and *** p<0.001.

ACE expressions were significantly higher in aortae of OVX rats than those of sham-operated rats as revealed by immunoblotting (Figure 4A) and immunohistochemistry (Figure 4B). The increased expression was normalized by chronic treatment with enalapril or valsartan (Figure 4A&B). Immunohistochemical staining showed an increased number of angiotensin II-positive cells in the vascular wall of the OVX rat aortas (Figure 4C). Chronic treatment with enalapril but not valsartan reduced the increased staining of angiotensin II (Figure 4C).

### AT_1_R and AT_2_R expression

**Figure 5:**
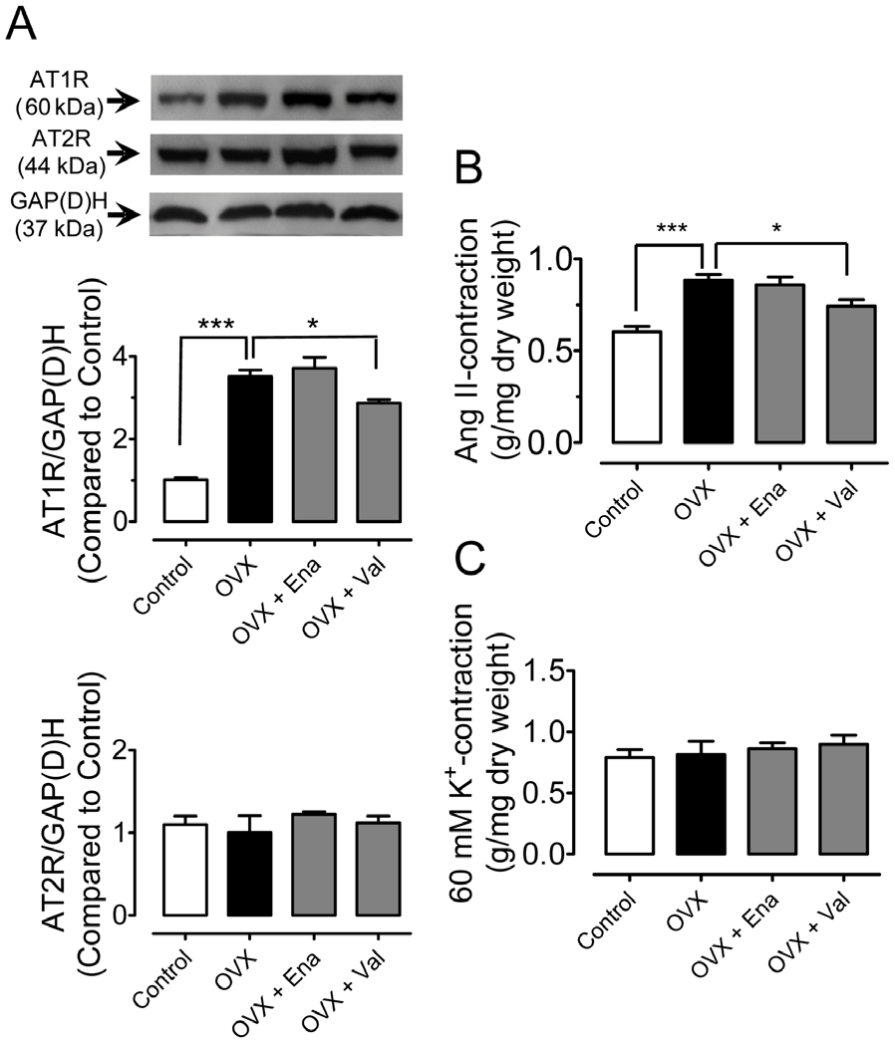
Chronic RAS blockade decreases AT_1_R expression. (A) Effects of chronic treatment with enalapril or valsartan on protein levels of angiotensin II type 1 (AT_1_R) and type 2 receptor (AT_2_R) in aortae of OVX rats. Aortic contraction induced by 100 nmol/l angiotensin II (B) and 60 mmol/l KCl (C) in the presence of 100 µmol/l L-NAME. Results are means±SEM of 4–5 experiments. Statistical significance is indicated by * p<0.05 and *** p<0.001.

The protein levels of AT_1_R were markedly elevated in aortae of OVX rats. Chronic treatment with valsartan but not enalapril reduced the up-regulation of AT_1_R expression (Figure 5A). By contrast, the protein expression for AT_2_R was unaltered by OVX or by drug treatment (Figure 5A). Angiotensin II (100 nmol/l) produced a greater contraction in OVX rat aortae and this augmented contraction was inhibited by chronic treatment with valsartan but not enalapril (Figure 5B). By contrast, 60 mmol/l KCl produced a comparable aortic contraction in different groups (Figure 5C).

### NAD(P)H oxidase expression and ROS production

**Figure 6:**
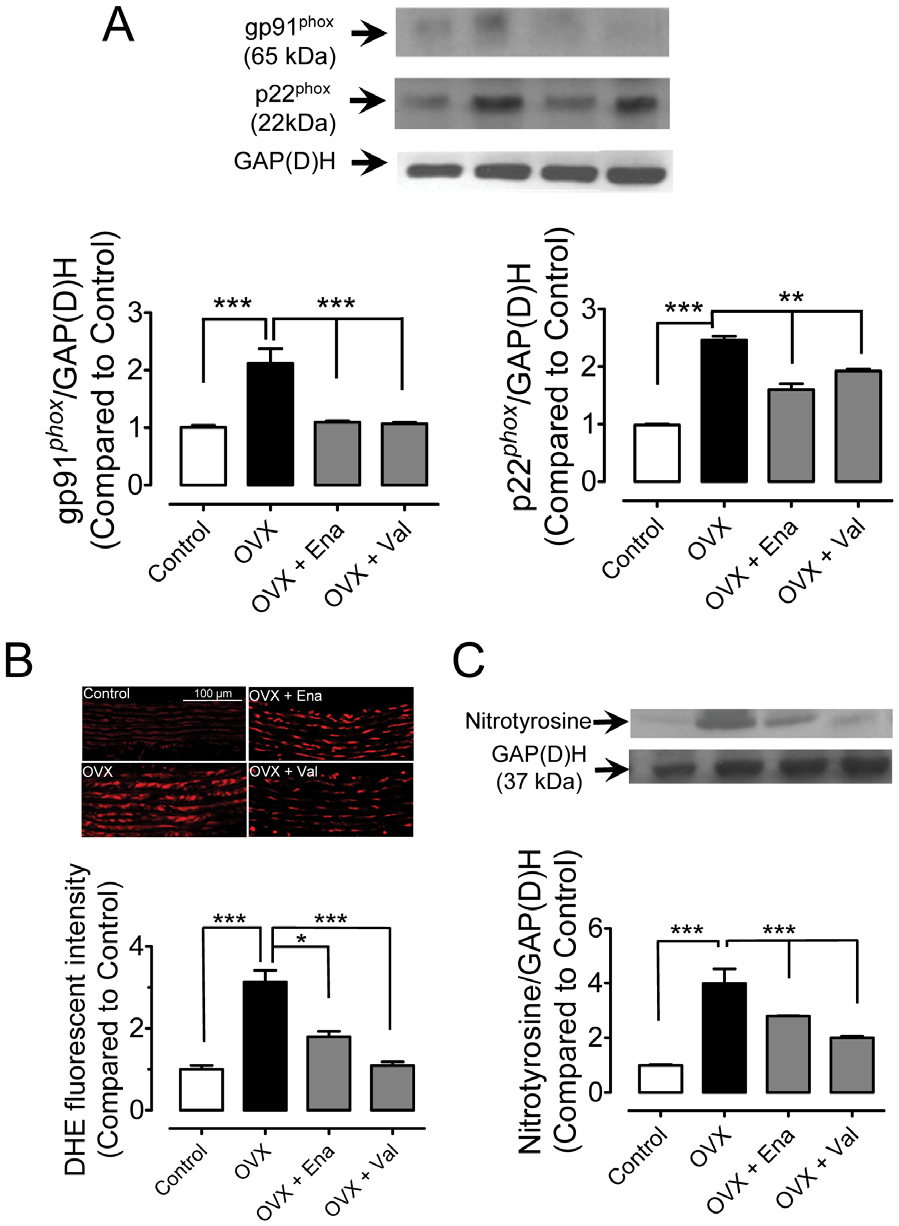
Chronic RAS blockade decreases oxidative stress. Effects of chronic treatment with enalapril or valsartan on the protein expression of NAD(P)H oxidase subunits: gp91*^phox^* and p22*^phox^* (A), on ROS production as revealed by DHE fluorescent intensity (B), and on the protein level of nitrotyrosine (C) in rat aortae. Results are means±SEM of 4–5 experiments. Statistical significance is indicated by ** p<0.01 and *** p<0.001.

Ovariectomy increased the expression of NAD(P)H oxidase subunits, gp91*^phox^* and p22*^phox^* in rat aortae, which was significantly inhibited in enalapril- or valsartan-treated OVX rats (Figure 6A). DHE fluorescence revealed an elevated ROS production in the vascular wall in the OVX rat aorta and this increase was attenuated by enalapril and prevented by valsartan treatment (Figure 6B). Likewise, the upregulation of nitrotyrosine, a marker of oxidative stress, in OVX rat aortae was largely inhibited by chronic treatment with both enalapril and valsartan (Figure 6C).

## Discussion

The present study reveals a principal role of the RAS activation and resulting oxidative stress in the induction of endothelial dysfunction in estrogen-deficient OVX rats. Endothelial dysfunction is caused by perturbation of the balance between NO and ROS. Ovariectomy leads to reduced NO bioavailability which accounts for the impaired endothelium-dependent relaxations during estrogen deficiency through a cluster of inter-connected cellular events in the vascular wall: (i) increased expression of ACE, (ii) augmented production of angiotensin II leading to over-expression of AT_1_R, (iii) greater production of ROS due to increased expression and activity of NAD(P)H oxidase, and (iv) the increased content of nitrotyrosine, a footprint for increased formation of peroxynitrite from NO and O_2_^−^. An important role for RAS activation in endothelial dysfunction was confirmed in OVX rats that were treated with valsartan and enalapril, widely used in the treatment of hypertension.

ROS are important contributors to endothelial dysfunction and subsequent development of vascular events. Increased ROS negates the vascular benefit of NO [22]. The present study shows that acute treatment with apocynin [a putative NAD(P)H oxidase inhibitor] markedly improves acetylcholine-induced relaxations of OVX rat aortae, suggesting a significant involvement of this oxidase in endothelial dysfunction. Furthermore, estrogen deficiency results in the increased expression of membrane-bound NAD(P)H oxidase submits, gp91*^phox^* and p22*^phox^*. The essential role of ROS is further supported by the observations that ROS scavengers, tiron plus DETCA, normalizes the impaired relaxations of OVX rat aortae. The present study also showed that the increased level of AT_1_R and augmented angiotensin II-induced vasoconstriction in the presence of L-NAME after ovariectomy was slightly but significantly inhibited to a similar extent by valsartan but not enalapril treatment. Angiotensin II stimulation of AT_1_R on the vascular wall is known to activate NAD(P)H oxidase [23], [24] and pathological roles of NAD(P)H oxidase are reported in hypertensive rats [13], [14]. In the present study, chronic RAS blockade blunts the increased expression of NAD(P)H oxidase in OVX rat aortae and this effect may be directly linked to the vascular benefit of RAS blockers. Increased oxidative stress in OVX rat aortae is further supported by elevated levels of nitrotyrosine, an oxidative stress biomarker. Staining intracellular ROS with DHE confirms over-production of O_2_^−^ in the vascular wall. Increases in both nitrotyrosine and ROS are inhibited or prevented by chronic RAS blockade. In addition, combined treatment with losartan and apocynin does not produce additional improvement in acetylcholine-induced relaxations, suggesting that both agents are equi-effective in reducing oxidative stress. Taken in conjuncture, AT_1_R-associated activation of NAD(P)H oxidase is the major stimulus for the over-production of ROS in OVX rat aortae and thus plays a key role in causing endothelial dysfunction during estrogen deficiency.

We next attempt to identify the possible triggering signal involved in increased oxidative stress during estrogen deficiency. Ovariectomy results in the increased expression of ACE in aortae, an effect that was not reported before. The increased levels of ACE would expectedly lead to an augmented production of angiotensin II. Indeed, higher levels of angiotensin II-positive cells in the vascular wall can be localized using immunohistochemical staining in OVX rat aortae. Chronic treatment with enalapril reverses the increased levels of ACE and angiotensin II to those in sham-operated rat aortae. Thus, the increased availability of angiotensin II likely accounts for the increased expression and activity of NAD(P)H oxidase that culminates in endothelial dysfunction. Previously studies reported that angiotensin II increased the protein levels of AT_1_R, NAD(P)H oxidase, and nitrotyrosine in cultured porcine endothelial cells [25] and that oxidative stress reduced the NO bioavailability in angiotensin II-infused mammals [26]. The present findings suggest a therapeutic potential of RAS inhibition in amelioration of oxidative stress-related endothelial dysfunction in states of estrogen deficiency.

ACE is also known to cleave bradykinin into biologically inactive smaller fragments and ACE inhibition preserves plasma bradykinin, a vasodilator in postmenopausal women [27]. If bradykinin contributes to the improved endothelial function, the augmented relaxations in enalapril-treated OVX rats would be blunted by bradykinin receptor blockade. Acute treatment with HOE-140, however, fails to produce such effects, suggesting that enalapril-induced endothelial cell protection is mediated primarily through inhibition of the synthesis of angiotensin II, rather than a decreased degradation of bradykinin in the vascular wall. Furthermore, bradykinin fails to relax rat aortae (data not shown), thus ruling out an involvement of bradykinin.

The present study provides novel evidence for complex mechanisms involved in the vascular benefit of valsartan. Analogous to the inhibitory effect of enalapril on the RAS-oxidative stress axis, valsartan exerts several previously un-reported benefits: (i) increasing the phosphorylation of eNOS at Ser^1177^ responsible for improved endothelial function, (ii) preventing the increased expression of ACE and thus reducing the over-production of angiotensin II, and (iii) reducing over-expression for AT_1_R. An elevated AT_1_R expression within the vascular wall of OVX rat aortae may be related to a loss of circulating estrogen as estrogen inhibits the basal expression and function of AT_1_R in cultured rat aortic vascular smooth muscle [4].

In the vascular wall, AT_1_R activation leads to unfavorable cellular responses including oxidative stress, lipid peroxidation, NO inactivation, and activation of redox-sensitive genes, which collectively participate in the initiation of vascular dysfunction [14], [22], [28]. The present study is probably the first to report that chronic valsartan treatment increases the NO bioavailability by increasing eNOS phosphorylation. Previous animal and human studies with AT_1_R blockers, however, did not determine the eNOS activity [29], [30]. Up-regulation of ACE, angiotensin II, and AT_1_R leads to the elevated expression and activity of NAD(P)H oxidase; subsequent over-production of ROS lowers the NO bioavailability and hence impairs endothelium-dependent relaxations. Reduced eNOS activity resulting from a loss of circulating estrogen [31] impairs endothelial function. However, it is yet to be determined whether there exists a difference in the long-term impact between early and late ovariectomy on vascular function due to possible difference in the duration of prior hormonal exposure in rats. This possibility deserves further examination. Treatment with valsartan or enalapril increased the plasma HDL level in OVX rats. In addition, valsartan treatment also lowered the non-HDL/HDL ratio in OVX rats and this may also benefit vascular function *in vivo*.

In summary, the present study elucidates a critical role of the RAS activation and associated oxidative stress in the induction and maintenance of endothelial dysfunction during estrogen deficiency. Treatment with enalapril (ACE inhibitor) or valsartan (AT_1_R blocker) restores endothelial function in OVX female rats. The novel findings provide mechanistic support for the limited clinical observations that RAS blockade may protect against cardiovascular diseases in postmenopausal women. Either ACE inhibitors or AT_1_R blockers could be an effective alternative to estrogen replacement or raloxifene therapy for menopausal women.

## Materials and Methods

### Drug treatment

This investigation conformed to the Guide for the Care and Use of Laboratory Animals published by the US National Institute of Health (NIH Publication No. 85-23, revised 1996), and approved by Animal Experimentation Ethics Committee of Chinese University of Hong Kong (4362/04 M). Adult female Sprague-Dawley rats (three-month old weighing 200–230 g) were supplied by the Animal Service Center of Chinese University of Hong Kong and housed under a 12-h light/dark cycle and fed *ad libitum*. Ovariectomy was performed via a mid-abdominal route after rats were anesthetized using sodium pentobarbital (40 mg kg^−1^ body weight via intraperitoneal injection) [10]. Four groups (6–8 rats in each) were included: (1) OVX rats receiving vehicle (OVX); (2) OVX rats receiving daily oral administration of 10 mg kg^−1^ enalapril (OVX+Ena) for eight weeks; (3) OVX+Val, OVX rats receiving daily oral administration of 10 mg kg^−1^ valsartan (OVX+Val) for eight weeks; and (4) sham-operated rats. The initial results showed that acetylcholine-induced relaxations were impaired starting at four weeks after ovariectomy and were maximally reduced (by 40%) twelve weeks after ovariectomy. Therefore, drug treatment was initiated at the 4^th^ week after surgery and lasted for the following eight weeks.

At the end of drug treatment, blood was drawn from the heart, collected in heparin-containing BD Vacutainer® while plasma was collected after centrifugation and stored frozen at −20°C until further bioassay. The concentration of plasma oestrogen was measured using immunosorbent assay kit (Cayman, Ann Arbor, MI, USA). After aortae were removed, the heart and uterus were dissected free of surrounding fat pads and weighed.

### Blood vessel preparation

The thoracic aorta was dissected and cleaned of adhering connective tissue in an ice-cold and oxygenated Krebs solution containing (mmol/l): 119 NaCl, 4.7 KCl, 2.5 CaCl_2_, 1 MgCl_2_, 25 NaHCO_3_, 1.2 KH_2_PO_4_, and 11 D-glucose. Each aorta was cut into several ring segments (∼3 mm long) for parallel studies. Each ring was suspended between two stainless steel hooks in a 10-ml organ bath filled with Krebs solution, which was bubbled with 95% O_2_ plus 5% CO_2_ and maintained at 37°C (pH: ∼7.4). One hook was fixed to the bottom of the bath while the other was connected to a Grass force displacement transducer. An optimal baseline tone of 2 g was applied to all rings. In some aortae, the endothelial layer was mechanically disrupted and functional removal of endothelium was verified by the lack of relaxation to 1 µmol/l acetylcholine. High KCl-containing solution was prepared by substituting NaCl with an equimolar amount of KCl to retain a constant ionic strength.

### Isometric tension measurement

Thirty minutes after setting up in organ bath, each ring was first contracted with 1 µmol/l phenylephrine and then relaxed by 1 µmol/l acetylcholine to test smooth muscle contractility and the integrity of the endothelium. Rings were then rinsed in pre-warmed Krebs solution until baseline tension returned. Two consecutive concentration-response curves to acetylcholine (3 nmol/l–10 µmol/l) were studied in control and in the presence (30-min incubation) of one of the following inhibitors: losartan (3 µmol/l), apocynin (100 µmol/l), tiron (1 mmol/l) plus diethyldithiocarbamate acid (DETCA, 100 µmol/l), and captopril (100 µmol/l). Acetylcholine-induced relaxations were compared in different treatment groups. Endothelium-independent relaxations to sodium nitroprusside (SNP) were also examined. Finally, a single concentration of angiotensin II (100 nmol/l) was used to trigger transient contraction in the presence of 100 µmol/L L-NAME because the aortae developed a rapid desensitization to consecutive application of angiotensin II. The contraction in response to 60 mmol/l KCl-containing Krebs solution was also performed and compared in aortae from different groups.

### Western blotting

Aortae were snap frozen in liquid nitrogen and homogenized in an ice-cold RIPA lysis buffer. The lysates were centrifuged and the supernatant was collected. The protein concentration was determined. Protein samples (50 µg) were separated with 10% SDS-polyacrylamide gel and then transferred to a nitrocellulose immobilon-P polyvinylidene difluoride membrane. The membranes were blocked with 1% bovine serum albumin. Primary antibodies against eNOS (1∶500, BD Transduction Laboratories, KY, USA), eNOS phosphorylated at ser^1177^ (1∶1000, Upstate Biotechnology, Lake Placid, NY), ACE (1∶1000, Santa Cruz, CA, USA), AT_1_R (1∶1000, Abcam, MA, USA), AT_2_R (1∶1000, Abcam, MA, USA), gp91*^phox^* (1∶500, Santa Cruz, CA, USA), p22*^phox^* (1∶500, Santa Cruz, CA, USA), nitrotyrosine (1∶1000, Upstate Biotechnology, NY, USA), and GAPDH (1∶3000, Ambion, TX, USA) were used. Corresponding secondary antibody conjugated to horseradish peroxidase (DakoCytomation) were used. The membranes were developed with an enhanced chemiluminescence detection system and exposed on X-ray films. Densitometry was performed using a documentation programme.

### Immunohistochemistry

Immunohistochemistry was performed on paraffin-embedded sections of aortic rings. Angiotensin II in the vascular wall was stained by rabbit polyclonal anti-angiotensin II antibody (1∶500 dilution in normal goat serum; Peninsula laboratory, CA, USA); and ACE by goat polyclonal anti-ACE antibody (1∶200 dilution in normal donkey serum; Santa Cruz, CA, USA), followed by secondary antibody conjugated with biotin (Jackson Immunoresearch, West Grove, PA, USA). Immunopositive signals were detected with streptavidin-HRP (Zymed, San Francisco, CA, USA), visualized with DAB (Vector laboratory, Burlingame, CA, USA), and counterstained with hematoxylin. The nonspecific binding was controlled by substitution of the primary antibody for negative comparison. Images were obtained under a light microscopy, and analyzed by ImageJ (NIH).

### ROS detection by dihydroethidium (DHE) fluorescence

Intracellular oxidants in aortic rings were measured using dihydroethidium (DHE; Molecular Probes, Eugene, OR, USA), which binds to DNA when oxidized to emit fluorescence. Frozen sections were cut in 15-µm thickness, and incubated at 37°C for 30 min in 5 µmol/l DHE. Fluorescence was observed under a confocal microscope (515-nm excitation; 585-nm long pass filter; Olympus Fluoview). DHE fluorescence intensity was analyzed by Fluoview (version 1.5; FV10-ASW1.5). For each section, a square region with an area of 80 µm×80 µm was selected for analysis. The summarized data represents the fold change in fluorescence intensity relative to that in control rat aortae.

### Chemicals

Phenylephrine, acetylcholine, sodium nitroprusside, captopril, apocynin, tiron, DETCA, N^G^-nitro-L-arginine methyl ester were from Sigma (Sigma-Aldrich, St. Louis, MO, USA). Losartan was purchased from Cayman Chemical (Ann Arbor, MI, USA). Apocynin and losartan were dissolved in dimethyl sulfoxide (DMSO) and others in distilled water. DMSO at 0.1% (v/v) did not affect acetylcholine-induced relaxations.

### Statistical analysis

Results represent means±SEM from n different rats. The concentration-response relationship was analyzed with a non-linear curve fitting (GraphPad Prism, Version 4.0). The p*D*_2_ was calculated as the negative logarithm of the dilator concentration that produced 50% of the maximum relaxation (E_max_). The protein expression was normalized to GAPDH and then expressed relative to the control. Student's *t*-test (unpaired two-tailed) was used and concentration-response curves were analyzed by two-way ANOVA followed by Bonferroni *post-hoc* tests. P<0.05 indicates significant difference.
